# Gender Differences in Online Health-Related Search Behaviors Among Older Adults

**DOI:** 10.1093/geroni/igab046.2484

**Published:** 2021-12-17

**Authors:** Matthew Picchiello, Payton Rule, Tina Lu, Brian Carpenter

**Affiliations:** 1 Washington University in St. Louis, University City, Missouri, United States; 2 Washington University in St. Louis, St. Louis, Missouri, United States; 3 Washington University in St. Louis, Saint Louis, Missouri, United States

## Abstract

Nearly 60% of older adults use the internet for health-related reasons. Some studies have demonstrated differences in the frequency at which men and women perform various online activities. However, few studies have investigated gender differences in health-related search behaviors (HRSB). The purpose of this study was to examine differences in self-reported HRSB between older men and women. A total of 47 older adults (M age = 66.6, 55% female, 87% White) completed a survey assessing perceived usefulness and trust in the internet for health-care information, types of websites used, and reasons for looking up health information. Independent samples t-tests revealed that, compared to women, men regard the internet as more useful in helping them make health care decisions (t (45) = 2.715) and as a more trustworthy source (t (45) = 2.24, p's < 0.05). Men were more likely to get health information through sources affiliated with educational institutions (χ2(1) = 3.9) and government agencies (χ2(1) = 8.8), whereas women were more likely to use social media, (χ2(1) = 4.3, p's < 0.05). Lastly, men were more likely to use the internet to learn about information on medical procedures (χ2(1) = 5.1), while women were more likely to learn about alternative treatments (χ2(1) = 4.9, p 's < 0.05) online. As 72.3% of participants indicated the internet as their first source for health information, interventions geared towards enhancing HRSB for older adults are needed, especially for older women whose HRSB may make them particularly vulnerable to misinformation.

